# Correction: Vitamin B3, nicotinamide, enhances mitochondrial metabolism to promote differentiation of the retinal pigment epithelium

**DOI:** 10.1016/j.jbc.2022.102493

**Published:** 2022-09-30

**Authors:** Roni A. Hazim, Antonio E. Paniagua, Lisa Tang, Krista Yang, Kristen K.O. Kim, Linsey Stiles, Ajit S. Divakaruni, David S. Williams

In the Experimental procedures of the original article (page 12, “Conditions for cell culture” section), the concentrations of stock solutions for taurine, hydrocortisone, and triiodothyronine were mistakenly reported rather than the final concentrations of those components in the medium. Additionally, nonessential amino acids (NEAA) were mistakenly omitted from the medium recipe.

Corrected text is provided below.

“Briefly, the cells were cultured in MEM-Nic (MEM alpha with GlutaMAX [Fisher Scientific]), 1% fetal bovine serum, 100 U/ml penicillin and 100 μg/ml streptomycin, 1% N2 supplement (Sigma–Aldrich), **taurine (0.25 mg/ml**) (Sigma–Aldrich), **hydrocortisone (20 ng/ml)** (Sigma–Aldrich), **triiodothyronine (0.013 ng/ml)** (Sigma–Aldrich), **0.1 mM NEAA (Fisher Scientific)**, and 10 mM nicotinamide (Sigma–Aldrich).”

The authors state that the corrections do not affect the results and conclusions of the paper.

